# Semantic Mapping Based on Spatial Concepts for Grounding Words Related to Places in Daily Environments

**DOI:** 10.3389/frobt.2019.00031

**Published:** 2019-05-28

**Authors:** Yuki Katsumata, Akira Taniguchi, Yoshinobu Hagiwara, Tadahiro Taniguchi

**Affiliations:** Department of Information Science and Engineering, Ritsumeikan University, Kyoto, Japan

**Keywords:** semantic mapping, spatial concept, Bayesian model, unsupervised learning, symbol emergence in robotics, ROS

## Abstract

An autonomous robot performing tasks in a human environment needs to recognize semantic information about places. Semantic mapping is a task in which suitable semantic information is assigned to an environmental map so that a robot can communicate with people and appropriately perform tasks requested by its users. We propose a novel statistical semantic mapping method called SpCoMapping, which integrates probabilistic spatial concept acquisition based on multimodal sensor information and a Markov random field applied for learning the arbitrary shape of a place on a map.SpCoMapping can connect multiple words to a place in a semantic mapping process using user utterances without pre-setting the list of place names. We also develop a nonparametric Bayesian extension of SpCoMapping that can automatically estimate an adequate number of categories. In the experiment in the simulation environments, we showed that the proposed method generated better semantic maps than previous semantic mapping methods; our semantic maps have categories and shapes similar to the ground truth provided by the user. In addition, we showed that SpCoMapping could generate appropriate semantic maps in a real-world environment.

## 1. Introduction

An autonomous robot performing tasks in our daily environment needs to recognize semantic information regarding the place. For example, when an autonomous vacuum cleaner robot tries to understand a command given by its user, e.g., “clean Joseph's room,” the robot needs to be able to locate “Joseph's room” on its map of the environment in order to clean that place. In addition, the places estimated by the robot need to have a region dealing with the shape of the environment. Semantic mapping is the task through which suitable semantic information is assigned to a robot's map so that it can communicate with people and appropriately perform tasks requested by its users (Kostavelis and Gasteratos, 2015).

**Figure 1:**
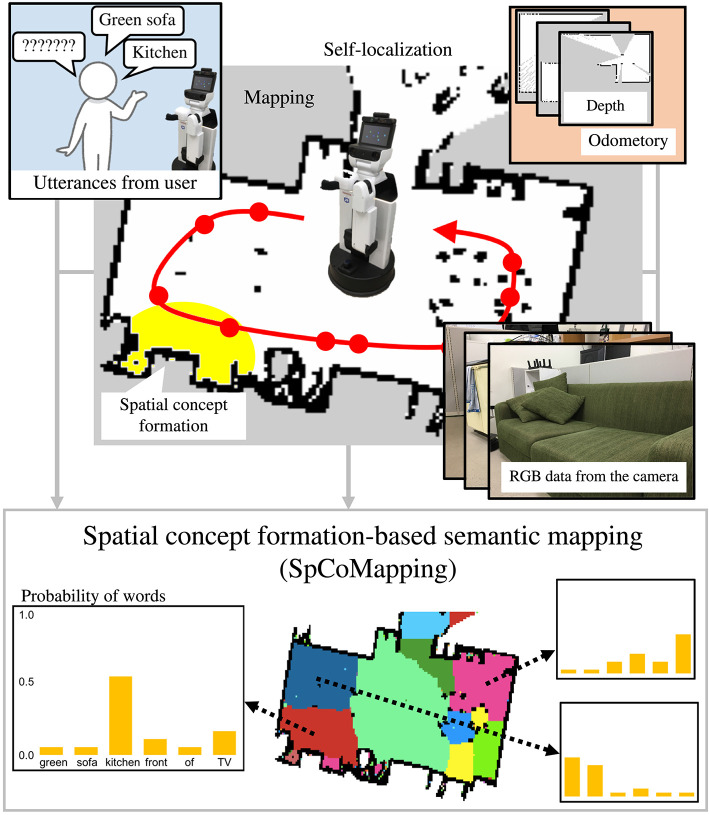
Overview of SpCoMapping. The robot moves around in the environment to obtain RGB data, words, and self-position data. It then learns spatial concepts by integrating multimodal information with a Markov random field and generates a semantic map.

Vocabulary used in daily human life depends on the environment a person is in, such as their home or office; a robot is unable to completely understand this, including words used to describe it, because the symbol system itself is a dynamic one (Taniguchi et al., 2016c). Many previous studies on semantic mapping (Kostavelis and Gasteratos, 2015; Goeddel and Olson, 2016; Sünderhauf et al., 2016; Himstedt and Maehle, 2017; Brucker et al., 2018; Posada et al., 2018; Rangel et al., 2019) have been conducted based on the assumption that a list of labels such as place names can be used as pre-existing knowledge; thus, they have been unable to estimate the meaning of place understood by a robot when is given a command including an unknown place name like “Joseph's room.” To deal with various environments by adapting semantically to them, and to collaborate with people, a semantic mapping method that can deal with unknown words uttered by users is crucial for service robots used in daily life. Therefore, this study proposes a novel statistical semantic mapping method called spatial concept formation-based semantic mapping (*SpCoMapping*) to address these issues. An overview of the SpCoMapping is shown in Figure 1.

Semantic mapping has been studied as a method to expand maps obtained using simultaneous localization and mapping (SLAM) into those including words. However, previous semantic mapping methods have three disadvantages.

1. The first is the overwrite problem, which is caused when the method overwrites the labels painted in previous cycles. For example, the image recognition results obtained when entering a room from the corridor and when leaving the room are different, even though the position is the same, because the visual images obtained by the robot are different in the two scenarios. Therefore, some methods overwrite the labels of the cells on the map that were generated on entering the room with new ones generated when the room is exited. However, this information should not merely be overwritten but should be stored statistically. Our proposed method solves this problem by modeling the room using semantic information from each cell on the map as a probabilistic variable.
2. Second, semantic maps generated by many previous methods are based solely on a single source of information, for example, depth or visuals. However, it can hardly be believed that people distinguish regions of a house semantically based on single sources of information. The regions and types of semantic categories on a semantic map formed in an environment should not only be influenced by one type information; it should also respond to multimodal information such as visual and location data, user utterances, and even other modalities such as sounds and smells. Our proposed method solves this problem by using a multimodal categorization method as part of the probabilistic generative model.
3. Third, many previous methods needed a list of place names to be set. However, we cannot expect all place names and features in our daily environment to be stored in the training dataset. For example, we cannot expect a training dataset generated for a typical house to include information on a particular person's room, e.g., “Joseph's room.” In contrast, our unsupervised learning method is based on a hierarchical Bayesian model that can acquire words related to places from sources such as user utterances. Therefore, it can obtain a vocabulary of words corresponding to a place along with their probability distributions.

A typical previous method is semantic mapping based on convolutional neural network (CNN) (Sünderhauf et al., 2016). Sünderhauf et al. (2016) proposed a method of semantic mapping with CNN that could convert RGB visual data into semantic labels. Image recognition results were used as semantic labels for mapping, and a robot painted the map generated by SLAM with the labels obtained by the CNN. This simple visual recognition-based approach also has the same problems.

Spatial concept formation methods have been developed to enable robots to acquire place-related words as well as estimate categories and regions (Ishibushi et al., 2015; Taniguchi et al., 2016b, 2017, 2018). These methods can estimate the number of categories using the Dirichlet process (Teh et al., 2005). Taniguchi et al. (2016b) proposed a nonparametric Bayesian spatial concept acquisition method (SpCoA). However, although spatial concept formation methods can acquire unknown words and deal with multimodal information, including the image features typically extracted using CNNs, they cannot perform semantically segment a map appropriately because the position distributions corresponding to semantic categories are modeled by Gaussian distributions. These methods cannot model the various shapes of regions on semantic maps. In our method, we adopt a Markov random field (MRF) to deal with various shapes on the semantic maps.

SpCoMapping integrates probabilistic spatial concept acquisition (Ishibushi et al., 2015; Taniguchi et al., 2016b) and SLAM via an MRF to generate a map of semantic information. It solves the overwrite problem by assigning each cell of the semantic map a probabilistic variable. It also deals with multimodal information using a multimodal categorization method as part of the probabilistic generative model. In addition, it does not need to set place names because we employ an unsupervised learning method that can acquire words related to places. Here, an unknown word implies that the word is not yet grounded in the map. In other words, the robot does not know the words related to a specific place on the map beforehand. Unknown word discovery from spoken sentences was performed in SpCoA (Taniguchi et al., 2016b); therefore, we obtained word information from sentences or words given in the experiment of this paper.

SpCoMapping has the following characteristics to solve the problems discussed above.

1. SpCoMapping can solve the overwrite problem.
2. Each region on the semantic map can have an *arbitrary shape*.
3. The semantic map is generated on the basis of also word information obtained through human-robot interactions as well as visual information, i.e., *multimodal information*.
4. SpCoMapping can relate *multiple words* to one place, without pre-setting the list of place names, using the semantic mapping process.
5. SpCoMapping can estimate the *number of semantic categories* using the Dirichlet process (Teh et al., 2005) as the prior distribution for semantic categories.

SpCoMapping was tested in two experiments, in simulation and in the real-world environment.

The remainder of this paper is organized as follows. Section 2 introduces existing semantic mapping and spatial concept acquisition methods. Section 3 describes our proposed method. Section 4 shows the results of the experiment conducted in a simulation environment. Section 5 shows the results of the experiment conducted by placing a robot in a daily human environment. Finally, section 6 concludes this paper.

## 2. Related Work

### 2.1. Semantic Mapping

The task of semantic mapping includes map segmentation and place recognition. Map segmentation is a task that categorizes places by hypothesizing that regions can be found by looking at the layout of free space (Fermin-Leon et al., 2017; Mielle et al., 2018; Tian et al., 2018). Mielle et al. (2018) proposed a method for segmenting maps from different modalities, which are able to use for robot-built maps and hand-drawn sketch maps. Place recognition is a challenging task for a robot; however, with CNNs and a large scene dataset, robots can understand places using image information (Guo et al., 2017; Xie et al., 2017; Xinhang et al., 2017; Wang et al., 2018b). Wang et al. (2018b) applied CNNs for omni-directional images for place recognition, and that result was used to allow robots to navigate.

Some studies on semantic mapping using two-dimensional (2D) maps such as topological maps (Garg et al., 2017; Liao et al., 2017; Pronobis and Rao, 2017; Luperto and Amigoni, 2018; Wang et al., 2018a) and occupancy grid maps (Goeddel and Olson, 2016; Sünderhauf et al., 2016; Himstedt and Maehle, 2017; Brucker et al., 2018; Posada et al., 2018; Rangel et al., 2019), were also conducted. Wang et al. (2018a) proposed a method that constructed a topological semantic map to guide object search. In addition, some studies attempted to provide methods that could correct topological semantic maps by mitigating the effects of noise or incorrect place recognition. Zheng et al. (2017, 2018) proposed a method that used graph-structured sum-product networks. They showed that this technique generates a semantic map from the results including incorrect nodes in place recognition. However, managing tasks like cleaning a room, which needs a place region, is difficult when a robot uses topological maps. Sünderhauf et al. (2016) employed a CNN to recognize place categories using visual information (i.e., RGB data) and laser-range data to build maps on which place categorization results are shown. They used the Places205 dataset (Zhou et al., 2014) to train the CNN, so that did not require environment-specific training. Unfortunately, this method could not deal with semantic information that was not included in the pre-existing training dataset.

In addition, three-dimensional (3D) semantic mapping in an indoor environment was studied in terms of performing tasks such as grasping or detecting an object simultaneously (Antonello et al., 2018; Li et al., 2018; Sun et al., 2018). Antonello et al. (2018) proposed a method that constructed a 3D semantic map online using the result of semantic segmentation. In addition, some studies assume that place categories are generated by objects in that environment and build semantic maps with object features (Stückler et al., 2015; Sünderhauf et al., 2017). Sünderhauf et al. (2017) proposed a method that built environment maps that included object-level entities and geometrical representations. They employed a single-shot multi-box detector (Liu et al., 2016) to detect objects and 3D SLAM to generate environment maps.

In this paper, we propose a method that generates 2D semantic maps. This is because 2D semantic mapping is challenging and a 2D semantic map can be applied to the autonomous vacuum cleaner robot.

### 2.2. Spatial Concept Formation

Taniguchi et al. (2016a) proposed a method that estimated words related to places and performed self-localization by Monte Carlo localization (MCL) simultaneously. Ishibushi et al. (2015) proposed a self-localization method that integrated semantic information obtained from image recognition performed by a CNN, following an idea proposed by Taniguchi et al. (2016a). Taniguchi et al. proposed SpCoA and an extension (Taniguchi et al., 2016b, 2018) that integrated a generative model for self-localization and unsupervised word segmentation in uttered sentences via the latent variables related to the spatial concept. However, all spatial concept acquisition methods assumed that the position information of each spatial region expressed a Gaussian distribution, i.e., that each semantic region had an ellipse-like shape. Therefore, these methods sometimes showed that the regions estimated based on the Gaussian distribution exceeded the area of a room. In contrast, SpCoMapping allows for arbitrarily shaped regions by adopting an MRF that takes the shape of the environment into account.

In addition, Taniguchi et al. (2017) proposed an online spatial concept acquisition and simultaneous localization and mapping (SpCoSLAM) method that integrates visual, position, and speech information and performs SLAM and lexical acquisition simultaneously. The complete learning process was performed online. Hagiwara et al. (2018) extended the spatial concepts as hierarchical categorizations. They showed that this method could acquire spatial concept hierarchically using vision, position, and word information. Our proposed method can also be appropriately extended online and hierarchically such as these methods.

## 3. Proposed Method

### 3.1. Overview

**Figure 2:**
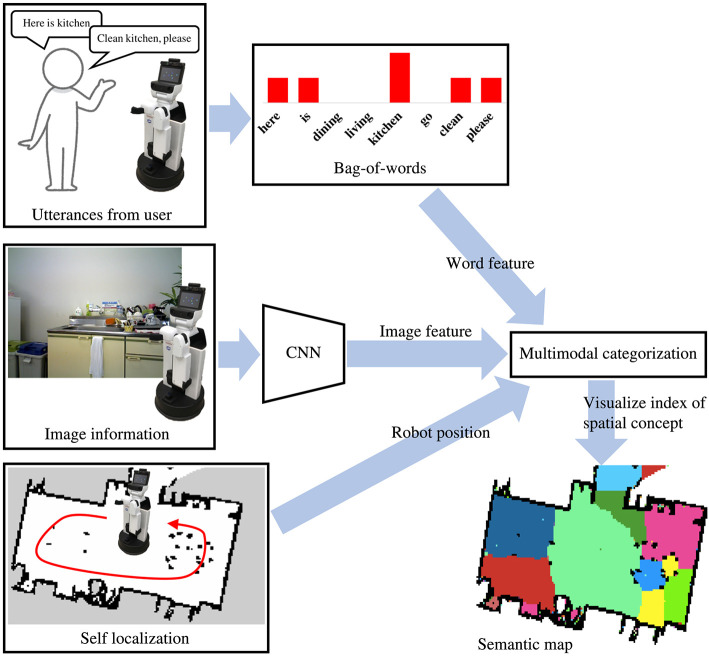
Flow diagram of SpCoMapping. The robot gets histograms of word features from bag-of-words information, histograms of image feature from CNN trained using the Places205 dataset (Zhou et al., 2014) and its position from the result of self-localization. We adopt word and image features and robot position for a multimodal categorization method and visualize indices of spatial concepts as a semantic map.

**Figure 3:**
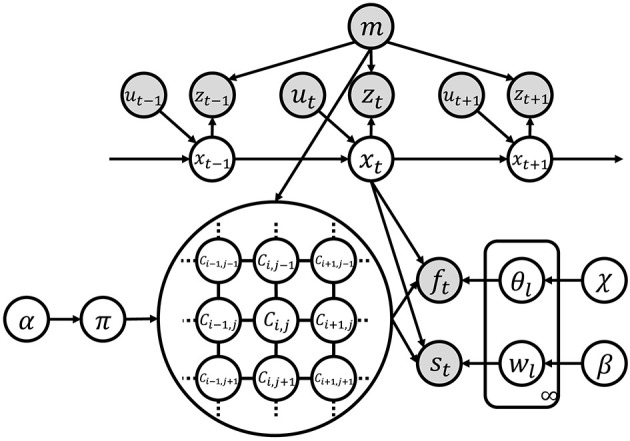
Graphical model of SpCoMapping. Gray nodes indicate observation variables and white nodes are unobserved variables.

The flow diagram of the process for the learning and semantic mapping is shown in Figure 2. The robot can create a map of the environment in advance using SLAM. The robot first moves around in an environment by self-localization using MCL and obtains RGB data. As it moves around, the user can talk to it by uttering the names of each place. In addition, SpCoMapping employs a pre-trained CNN, similar to that in Sünderhauf et al. (2016), to obtain a probability distribution of the place labels to use as a feature vector of the proposed probabilistic generative model. The speech signals uttered by the user are recognized by a speech recognition system, and the results are provided to SpCoMapping as word information. We adopted bag-of-words (BoW) as word information because the count of the words uttered in each place represents a word feature. The robot next learns the spatial concepts by integrating multimodal data and generates a semantic map using the probabilistic generative model shown in Figure 3.

The pseudo-code of SpCoMapping is shown in Algorithm 1. The procedure of the method is described as follows:

1. The robot initializes *C*_*i, j*_ described by MRF from the occupancy grid map. *I* and *J* represent the width and height of the map, respectively (line 2 in Algorithm 1).
2. If a pixel on the occupancy grid map does not correspond to free space, then there are no spatial concepts in this model, and the robot retains the *C*_*i, j*_ value (line 6–7).
3. The robot converts *x*_*t*_, which denotes the coordinates obtained by MCL, to (*i, j*), which denotes the pixel on the occupancy grid map. The equation is shown in (11) (line 9).
4. For every free space on the occupancy grid map, the robot obtains an index of the spatial concepts by sampling (line 10–12).
5. The robot uses sampling to obtain the multinomial distribution of the index of spatial concepts (line 16).
6. For each spatial concept category, the robot uses sampling to obtain the multinomial distribution of image features (line 18).
7. For each spatial concept category, the robot uses sampling to obtain the multinomial distribution of vocabulary features (line 19).

**Algorithm 1:** *Semantic mapping based on spatial concepts*

1: initializationπ, θ, *w*
2: get*C*_(1:*I*, 1:*J*)_frommap
3: **for** *h* = 1 **to** *iteration* **do**
4: **for** *i* = 1 **to** *I* **do**
5: **for** *j* = 1 **to** *J* **do**
6: **if** *C*_*i, j*_isoccupied **then**
7: keep*C*_*i, j*_value
8: **else**
9: convert*x*_*t*_to(*i, j*) //Equation (11)
10: $t^{\prime }\in \left\{t|C_{\text{convert}\left(x_{t}\right)}=l,t\in \left(1:T\right)\right\}$
11: *C*_*i, j*_~MRF(*C*_*i, j*_∣*C*_∂(*i, j*)∣*m*_; γ)Mult(*C*_*i, j*_∣π)
12: $\times \prod_{t^{\prime }}\left[\text{Mult}\left(s_{t^{\prime }}|x_{t^{\prime }},w_{C_{i,j}}\right)\text{Mult}\left(f_{t^{\prime }}|x_{t^{\prime }},\theta_{C_{i,j}}\right)\right]$
13: **end if**
14: **end for**
15: **end for**
16: $\pi \sim \text{Dir}\left(\pi |\overset{I}{\underset{i=1}{\sum }}\overset{J}{\underset{j=1}{\sum }}C_{i,j}+\frac{\alpha }{L}\right)$
17: **for** *l* = 1 **to** *L* **do**
18: $\theta_{l}\sim \text{Dir}\left(\theta_{l}|\underset{t^{\prime }}{\sum }f_{t^{\prime }}+\chi \right)$
19: $w_{l}\sim \text{Dir}\left(w_{l}|\underset{t^{\prime }}{\sum }s_{t^{\prime }}+\beta \right)$
20: **end for**
21: **end for**
22: save*C*, π, θ_*l*_, *w*_*l*_

The details of the sampling process are described in section 3.3.

### 3.2. Definition of Generative Model and Graphical Model

Figure 3 shows the graphical model of SpCoMapping, and Table 1 shows the list of variables in the graphical model. We describe the probabilistic generative process represented by the graphical model as follows:

(1) π~DP(α)

(2) $$C_{i,j}\sim p(C_{\partial (i,j)},\;\pi ,\;m)$$

(3) $$\theta_{l}\sim \text{Dir}(\chi )$$

(4) $$w_{l}\sim \text{Dir}(\beta )$$

(5) $$f_{t}\sim \text{Mult}(x_{t},\theta_{C_{i,j}})$$

(6) $$s_{t}\sim \text{Mult}(x_{t},w_{C_{i,j}})$$

where ∂(*i, j*) represents the neighborhood pixels of the (*i, j*) pixel, *C*_∂(*i, j*)_ represents the neighborhood node of *C*_*i, j*_, Dir represents the Dirichlet distribution, and Mult represents the multinomial distribution.

**Table 1:** Definition of variables in the graphical model.

*m*	Map of environment
*x*_*t*_	A robot's self-position information
*u*_*t*_	Control information
*z*_*t*_	Distance information
*C*_*i, j*_	Index of spatial concept in (*i, j*) pixel
*f*_*t*_	Image feature
*s*_*t*_	Vocabulary feature (bag-of-words)
π	Parameter of multinomial distribution for *C*_*i, j*_
θ_*l*_	Parameter of multinomial distribution for *f*_*t*_
*w*_*l*_	Parameter of multinomial distribution of *s*_*t*_
α, β, χ	Hyperparameters of prior distributions

In (1), DP represents the Dirichlet process (DP). DP is a probabilistic process that can generate the parameters of an infinite-dimensional multinomial distribution. A nonparametric Bayesian clustering method that uses DP can automatically estimate the number of clusters. We adopted weak-limit approximation for calculating DP (Fox et al., 2011), described as:

(7) $$\text{DP}(\alpha )\approx \text{Dir}\left(\frac{\alpha }{L},\ldots ,\frac{\alpha }{L}\right)$$

where *L* is the upper limit of the spatial concepts.

In (2), MRF represents the MRF described in the same way as in Chatzis and Tsechpenakis (2010). The equation is as follows:

(8) $$\text{MRF}(C_{i,j}|C_{\partial (i,j)};\gamma )\propto \exp\left\{\sum_{r\in \partial (i,j)}\gamma \delta (C_{i,j},C_{r})\right\}$$

(9) $$\delta (a,b)=\{\begin{array}{l} 1\;(a=b)\\ 0\;(a\neq b) \end{array}$$

(10) $$p(C_{\partial (i,j)},\;\pi ,\;m)=\text{MRF}(C_{i,j}|C_{\partial (i,j)|m};\gamma )\text{Mult}(C_{i,j}|\pi )$$

where γ represents the temperature parameter and *C*_∂(*i, j*)∣*m*_ means that *C*_∂(*i, j*)_ is generated from the occupancy grid map *m*.

In (5) and (6), *x*_*t*_ is the self-position of a robot and (*i, j*) represents the 2D index of pixels on the occupancy grid map. *f*_*t*_ and *s*_*t*_ are sampled only if self-position *x*_*t*_ corresponds to the (*i, j*) pixel. The equation to convert *x*_*t*_ to (*i, j*) is

(11) $$\begin{array}{l} \left(i,j)=\text{convert}(x_{t}\right)\\ \;=\left\lfloor \frac{x_{t}-X}{k}\right\rfloor \end{array}$$

where ⌊(*p*_*x*_, *p*_*y*_)⌋ represents the floor function. This equation means that the maximum integer coordinates of the x-axis are not greater than real number *p*_*x*_ and those of the y-axis are not greater than real number *p*_*y*_. *X* represents the original pose of a robot, and *k* represents the size of one pixel on the map in a real environment. The origin pose refers to the coordinates of the (1, 1) pixel. We obtained both using the *occupancy grid message* of the robot operating system (ROS) (Quigley et al., 2009).

### 3.3. Details of the Sampling Procedure

SpCoMapping estimates *C*_*i, j*_, π, θ_*l*_, and *w*_*l*_ using Gibbs sampling. The procedure for each sampling is shown below.

- Sampling *C*_*i, j*_:
     If *m*_*x*_*t*__ is not free space, then *C*_*i, j*_ have no spatial concept.

(12) $$C_{i,j}\text{= 0}$$

If *m*_*x*_*t*__ is free space, then the sampling equation is

(13) $$\begin{array}{l} \;t^{\prime }\in \{t|C_{\text{convert}(x_{t})}=l,t\in (1:T)\}\\ C_{i,j}\sim \text{MRF}(C_{i,j}|C_{\partial (i,j)|m};\gamma )\text{Mult}(C_{i,j}|\pi )\\ \;\times \prod_{t^{\prime }}\left[\text{Mult}(s_{t^{\prime }}|x_{t^{\prime }},w_{C_{i,j}})\text{Mult}(f_{t^{\prime }}|x_{t^{\prime }},\theta_{C_{i,j}})\right] \end{array}$$

where *t*′ represents an element of the set of times when *C*_*i, j*_ = *l* in the converted self-positions (*i, j*) = convert(*x*_*t*_) (*t*∈(1:*T*)). *T* is the number of training data.

- Sampling π:
     When the quantities of spatial concepts are unknown, we adopt DP. The sampling equation is shown in (14).

(14) $$\pi \sim \prod_{i=1}^{I}\prod_{j=1}^{J}\text{Mult}(C_{i,j}|\pi )\text{DP}(\pi |\alpha )$$

When the quantities of spatial concepts are known, we adopt Dirichlet distribution. The sampling equation is

(15) $$\pi \sim \prod_{i=1}^{I}\prod_{j=1}^{J}\text{Mult}(C_{i,j}|\pi )\text{Dir}(\pi |\alpha )$$

- Sampling θ_*l*_ and *w*_*l*_:
     The sampling equations are

(16) $$\theta_{l}\sim \prod_{t^{\prime }}\text{Mult}(f_{t^{\prime }}|x_{t^{\prime }},\theta_{C_{i,j}})\text{Dir}(\theta_{l}|\chi )$$

(17) $$w_{l}\sim \prod_{t^{\prime }}\text{Mult}(s_{t^{\prime }}|x_{t^{\prime }},w_{C_{i,j}})\text{Dir}(w_{l}|\beta )$$

Finally, SpCoMapping can infer the semantic category of each pixel on the occupancy grid map using Gibbs sampling. The semantic mapping is achieved by this inference.

## 4. Experiment 1: Simulation Environment

**Figure 4:**
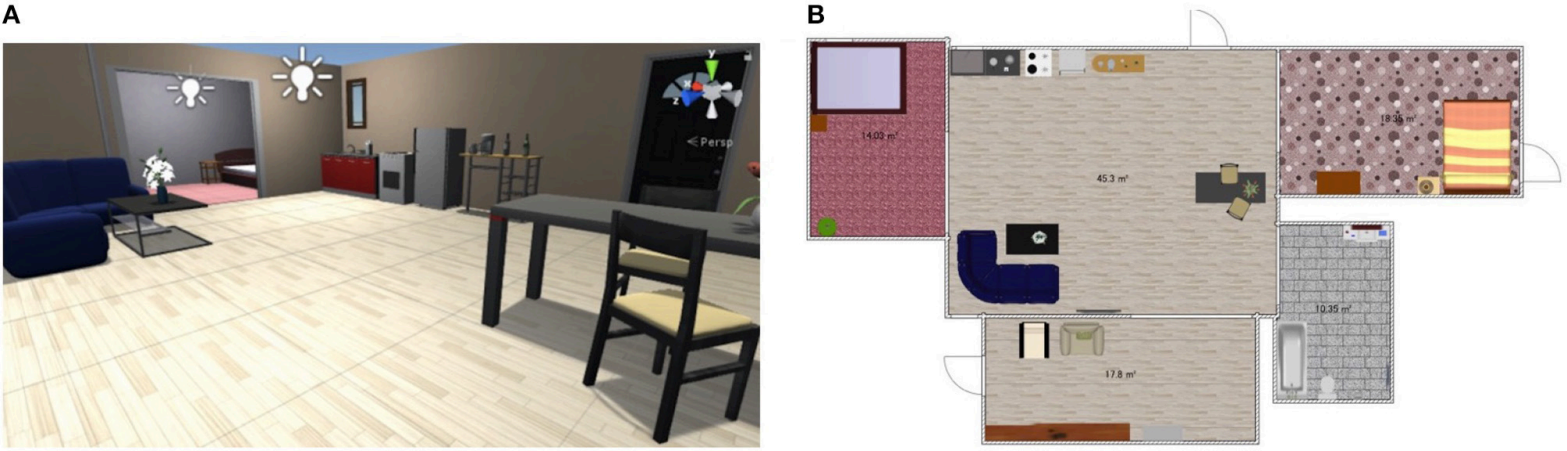
Example of the simulation environment. **(A)** example image of the SIGVerse and **(B)** example map of the experiment in the simulation. Both of them are the example of Room2ldk4.

We experimented to evaluate the semantic mapping ability of SpCoMapping and compare it with that of existing methods. For the quantitative evaluation, we performed experiments in the simulation environment SIGVerse^1^ that emulated the daily living environment. We have provided the source code^2^ for SpCoMapping and the test dataset^3^ used in this experiment for public access on Github. Figure 4 shows the environment used in our experiment in SIGVerse. Table 2 presents information on the rooms in the simulation environment.

**Table 2:** Information about each room in the simulation.

**Environment**	**Pixels**	**Categories on ground truth**
Room1dk5	18622	6
Room1dk6	26254	5
Room1ldk4	18026	7
Room1ldk5	22139	7
Room2ldk4	27746	7

### 4.1. Conditions

We employed Caffe (Jia et al., 2014)—a deep learning framework—to implement the CNN. To train the CNN, we used the Places205 dataset (Zhou et al., 2014) and used AlexNet as the particular network architecture for the CNN (Krizhevsky et al., 2012). To give word information to the robot, we provided it with textual place name data directly, without using speech recognition, to keep the focus on evaluating the semantic mapping. We compared the following methods:

- SpCoMapping with DP prior (without knowledge of the number of semantic categories)
- SpCoMapping with Dirichlet prior (with knowledge of the number of semantic categories)
- Spatial concept formation with word information (Ishibushi et al., 2015)
- CNN-based semantic mapping of Sünderhauf et al. (2016)
- Nearest neighbor.

For method (A), we set the upper limit for spatial concepts to 120. For method (B), we set the number of spatial concepts to be the same as the number of ground truths. Ishibushi et al. (2015)'s method only employed image features and self-localization. However, in method (C) in our experiment, we compared it to a model designed to handle word information. Method (D) does not categorize all pixels on the occupancy grid maps; therefore, we adopted the nearest neighbor for this method to fill up all the pixels for the comparisons. Method (E), the nearest-neighbor method, is one of the easiest: it retrieves a word label from the sample nearest to its position.

We prepared the ground truth labels by asking a participant to draw a semantic map for each map by referring to the information from the 3D simulator and the 2D maps. In the simulation, we gave the robot words that were assumed to be used in each environment. The vocabulary list is summarized in Table 3 and includes underlined words that are not labels of the Places205 dataset.

**Table 3:** Vocabulary list used in the simulation environment.

**Bedroom**	**Shower**	**Kitchen**	**Entrance**	**Living**
Dining	Closet	Corridor	Parlor	
Window	Tree	Shelf	Chair	Sofa
Washroom	Toilet	Refrigerator	TV	Wall
Bed	Table	Bath	Door	

*Underlined words are not included in the Places205 dataset*.

We performed 5,000 iterations of SpCoMapping for methods in which the number of spatial concepts are both unknown and known. We set the hyperparameters for DP as follows: α = 1.0 * 10^8^, β = 500, χ = 1.0 * 10^4^, and γ = 4.0. We set the hyperparameters for Dirichlet distribution as follows: α = 5.0 * 10^7^, β = 100, χ = 1.0 * 10^4^, and γ = 3.0. We used the following hardware and middleware: Ubuntu 14.04 LTS 64-bit, ROS Indigo with 31.4 GB memory, Intel Core i7-4770K CPU @ 3.50 GHz 8, and Gallium 0.4 on NVE4.

### 4.2. Results

#### 4.2.1. Clustering Accuracy

We calculated the adjusted Rand index (ARI) (Hubert and Arabie, 1985), which is a measure of similarity between two clusters, for each method. If two clusters are the same, the ARI is one; if each cluster are allocated randomly, the ARI is zero. Semantic mapping can be regarded as a task in which pixels are clustered on a map. We compared the performance of the methods from this viewpoint.

**Figure 5:**
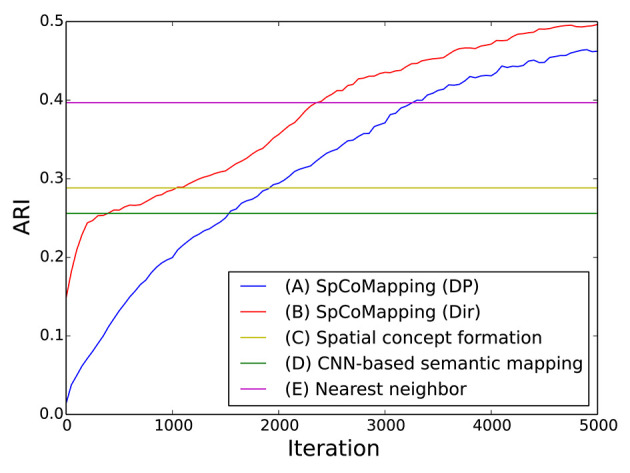
Example of the changes in the ARI by iteration (Room2ldk4).

**Figure 6:**
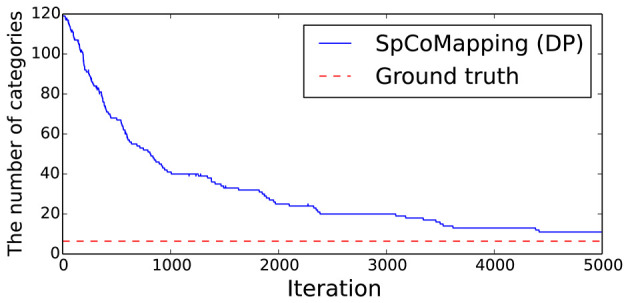
Example of the changes in the number of categories by iteration on the proposed method (Room1ldk4).

**Figure 7:**
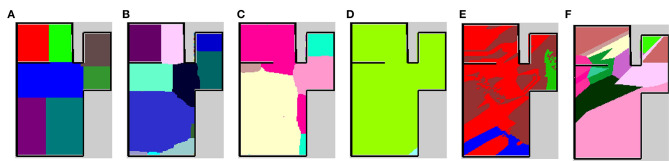
Semantic maps of the experiment in simulation environment Room1ldk4. **(A)** The map of ground truth drawn manually, **(B)** the map of SpCoMapping when the number of spatial concepts are unknown, **(C)** map of SpCoMapping when the number of spatial concepts are known, **(D)** map of the existing spatial concept acquisition method (Ishibushi et al., 2015), **(E)** map of the CNN-based semantic mapping (Sünderhauf et al., 2016), and **(F)** map of nearest neighbor. The colors represent different categories on each map.

The results are shown in Table 4a. The column titled “Average” denotes the average ARI of the five rooms. SpCoMapping has a higher average, showing a higher performance on each map, compared to the other methods. This result suggests that SpCoMapping can solve the problems introduced in section 1, including the overwrite and shape problems; in other words, the categories of semantic maps it generates are closer than the other semantic mapping methods to the categories of semantic maps generated by a person. In this result, SpCoMapping with DP prior is better than SpCoMapping with Dirichlet prior which the number of categories is given. As same as (Nakamura et al., 2015) when Gibbs sampling algorithm samples using fixed quantities of categories, it is sometimes harder than using changing quantities of categories. Figure 5 shows an example of the change in the ARI by iteration for Room2ldk4. The increasing iterations also increased the ARI. Figure 6 shows an example of the change in the categories of Room1ldk4 caused by iterations of SpCoMapping (DP). This result shows that SpCoMapping (DP) gradually estimated the number of semantic categories. However, the relationship between the iteration and the number of categories depends on the size of the map and the complexity of the environment. Therefore, in future work, we need to improve the ability to automatically determine the number of iterations. Figure 7 shows the semantic maps generated by each method. The regions on the maps generated by SpCoMapping are separated on the wall and do not put the wall between the regions itself. These maps show that the places estimated by SpCoMapping have regions dealing with the shape of the environment.

**Table 4:** The results of simulation environment.

**Method**	**Room1dk5**	**Room1dk6**	**Room1ldk4**	**Room1ldk5**	**Room2ldk4**	**Average**
**(a) The results of ARI**						
(A) SpCoMapping (DP)	**0.5356**	0.4548	**0.5877**	**0.4925**	0.4623	**0.5066**
(B) SpCoMapping (Dir)	0.4471	**0.5355**	0.4393	0.3354	**0.4963**	0.4507
(C) Spatial concept formation	0.3524	0.4692	0.2657	0.3740	0.2883	0.3499
(Ishibushi et al., 2015)						
(D) CNN-based semantic mapping	0.3102	0.2742	0.2505	0.3067	0.2559	0.2795
(Sünderhauf et al., 2016)						
(E) Nearest neighbor	0.3371	0.2870	0.4830	0.3337	0.3968	0.3675
**(b) Results of the matching rates for place estimation**						
(A) SpCoMapping (DP)	**0.2612**	0.0268	**0.2185**	0.1338	0.0001	0.1106
(B) SpCoMapping (Dir)	0.2071	**0.6368**	0.1461	0.1119	0.1780	**0.2715**
(C) Spatial concept formation	0.0463	0.2587	0.2154	**0.3200**	**0.1876**	0.2113
(Ishibushi et al., 2015)						
(D) CNN-based semantic mapping	0.1174	0.0761	0.0949	0.0766	0.1245	0.0979
(Sünderhauf et al., 2016)						
(E) Nearest neighbor	0.1260	0.1607	0.1729	0.1252	0.1292	0.1422

*Bold number is the best in that environment and underlined number is the two best*.

#### 4.2.2. Estimation of Place by a Word Input

When a robot is required to perform a task that requires communication with the user, e.g., navigation, cleaning the room, or searching for an object, the robot needs to estimate the place indicated by the user from a word input. Therefore, we compared the matching rate of the places estimated by each method using the following calculations:

(18) $$R_{match}=\sum_{s\in V}^{\left|V\right|}\frac{M_{\left\{m_{C_{s}}=m_{L_{s}}\right\}}}{M_{free\_space}}$$

where *V* represents vocabulary, i.e., the set of words, in the ground truth; *M*_*condition*_ represents the number of spaces which meet the condition. *m*_*C*_ is the pixels on the semantic map or ground truth given category *C*. *L*_*s*_ represents the category of ground truth given word *s*. The equation for estimating an index of spatial concepts from the word *s* inputs is as follows:

(19) $$C_{s}=\underset{C}{\text{argmax}}\;p(C|s_{t}=s,\;\pi ,\;w)$$

The results are shown in Table 4b. Method (B) performs better, with a score of “average,” compared to the other methods. This result shows that SpCoMapping can estimate place regions better than previous methods when the robot is given a command by its user that includes the place name. Method (A) performed the best in Room1dk5 and Room1dk4. However, it did not have good results for a large environment (Room1dk6 and Room2ldk4). The reason for the poor performance of SpCoMapping in the two large environments is attributed to the creation of many clusters for a large region and a wrong estimate of the number of categories in these environments. The nonparametric Bayesian estimation of semantic categories is unstable, as shown in the result. SpCoMapping (Dir) is more stable than SpCoMapping (DP).

## 5. Experiment 2: Real-World Daily Environment

### 5.1. Conditions

**Figure 8:**
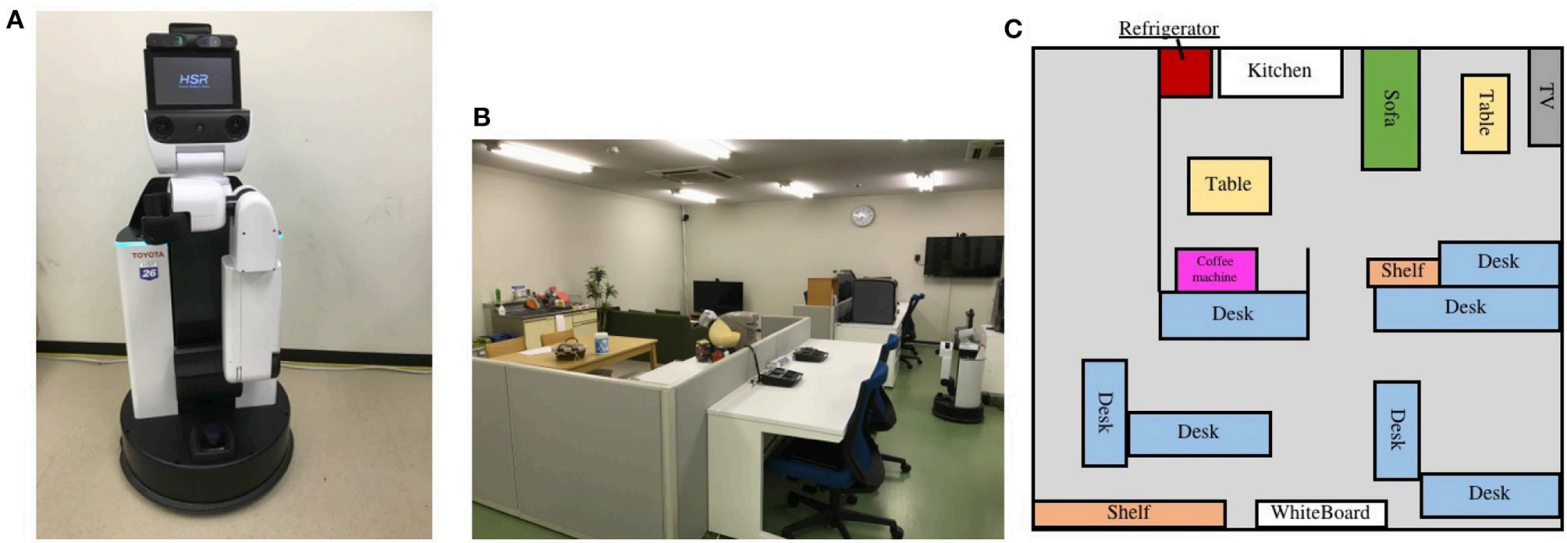
The robot and the example of experiment on the real environment. **(A)** Robot employed for experiments on the personal living environment. We used the Human Support Robot (HSR) from TOYOTA; it has Xtion PRO LIVE by ASUS to obtain RGB data and UST-20LX by Hokuyo to obtain laser-range data. Ubuntu 16.04 and ROS kinetic were installed on the PC. **(B)** The image of the environment employed in this experiment and **(C)** the map of the environment.

We experimented to generate a semantic map in a real-world environment. The robot and environment we employed are shown in Figure 8, respectively. The laboratory room serves as the living environment for experiments and as a study space for the researcher. In this experiment, we obtained word information from given sentences to demonstrate that SpCoMapping can acquire vocabularies as place names without setting them. We used sentences as word features to show how multiple words could be connected to a place without pre-setting the place names. The sentence list is shown in Table 5. We provided these 20 sentences, which include 50 vocabularies, five times for each sentence. We provided the RGB data 407 times.

**Table 5:** Sentence list used in the real-world daily environment.

Here is kitchen
Please take cup on kitchen
Bottle is on dining table
Here is dining
Here is living space
TV and sofa are in living space
Put remote controller on living table
You can use desk in living space
Can you find dishes on kitchen
You can use coffee machine in dining
Refrigerator is near kitchen and dining
Do not clash with sofa when you are in kitchen
Here is meeting space
Whiteboard is in meeting space
Here is study space
Please clean study space
They are holding meeting now
Please gather around whiteboard we will start meeting
Here is study room too
You can use desk in study space

For this experiment, we adopted SpCoMapping (DP) when the quantities of spatial concepts were unknown. We set the upper limit number of spatial concepts to 120. We set the hyperparameters as follows: α = 1.0 * 10^6^, β = 0.6, χ = 100.0, and γ = 4.0.

We set the weight for vocabulary feature using the tf-idf scheme (Salton and Mcgill, 1986) as mutual information between words and sentences. The equation used to calculate the weight for word *i* in sentence *j* is as follows:

(20) $${\text{Weight}}_{i,j}=\frac{n_{i,j}}{\sum_{k}n_{k,j}}\log\frac{D}{D_{i}}$$

where *n*_*i, j*_ is the number of words *i* in sentence *j*, *D* is the number of sentences, and *D*_*i*_ represents the number of sentences including word *i*. By setting the weight for words using tf-idf, the importance of words included in many sentences, for example, “is,” “here,” and “you,” are lower. This process helps in the acquisition of place-related words.

In order to ensure the stability of the proposed method, we sample *w*_*l*_ for 100 times and use the average as *w*_*l*_.

In addition, we employed pre-learning by spatial concept formation using word information (Ishibushi et al., 2015). We set hyperparameters as the same parameters and calculated 1000 iterations. In Algorithm 1 line 1, we initialized π, θ, *w* using the result of pre-learning. In Algorithm 1 line 2, we initialized *C*_*i, j*_ as follows: If *m*_*x*_*t*__ is not a free space, then *C*_*i, j*_ have no spatial concept.

(21) $$C_{i,j}=\text{0}$$

**Figure 9:**
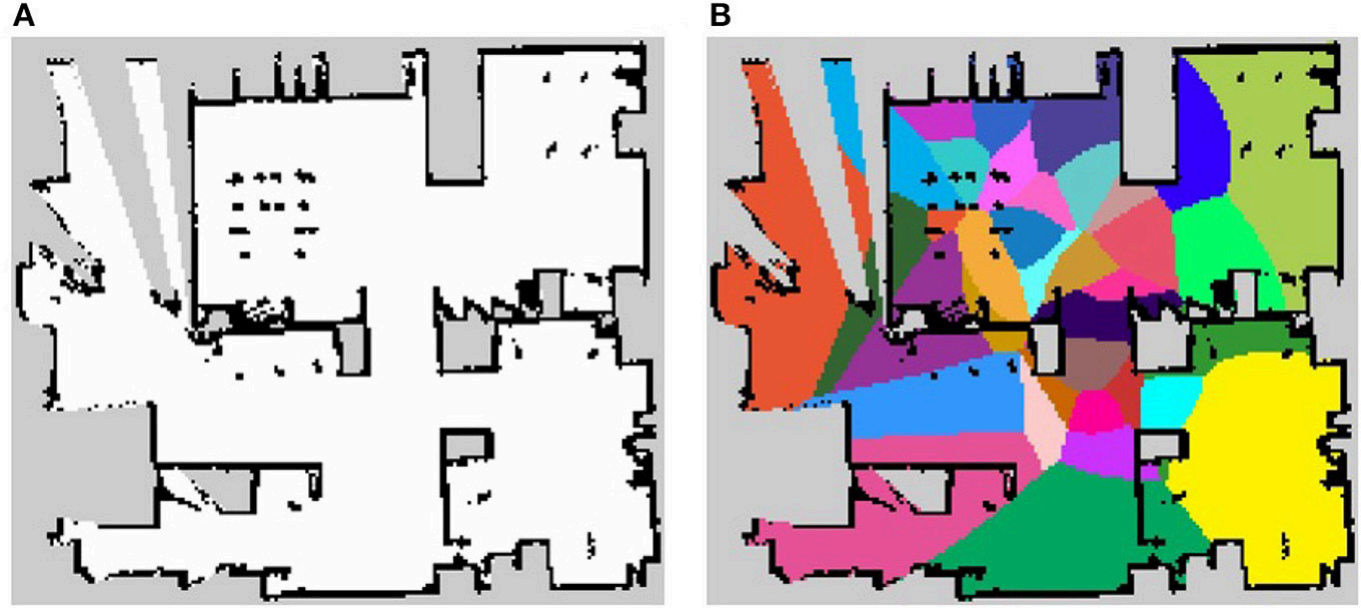
The maps used in the experiment. **(A)** The occupancy grid map of personal living environment and **(B)** the result of pre-learning.

If *m*_*x*_*t*__ is a free space, then the sampling equation is as follows:

(22) $$C_{i,j}=\underset{l}{\text{argmax}}\;N((i,j)|\mu_{l}^{pre},\;\Sigma_{l}^{pre})$$

where the multivariate Gaussian (normal) distribution is N, $\mu_{l}^{pre}$ is the mean vector of position distribution on the *l*-th pre-learning category, and $\Sigma_{l}^{pre}$ is the covariance matrix of the position distribution on the *l*-th pre-learning category. When pre-learning is employed vocabulary acquisition features are more stable and fewer iterations are required for learning. The occupancy grid map of the personal living environment and the result of pre-learning are shown in Figure 9. This occupancy grid map has 19,255 pixels as free space.

### 5.2. Results

**Figure 10:**
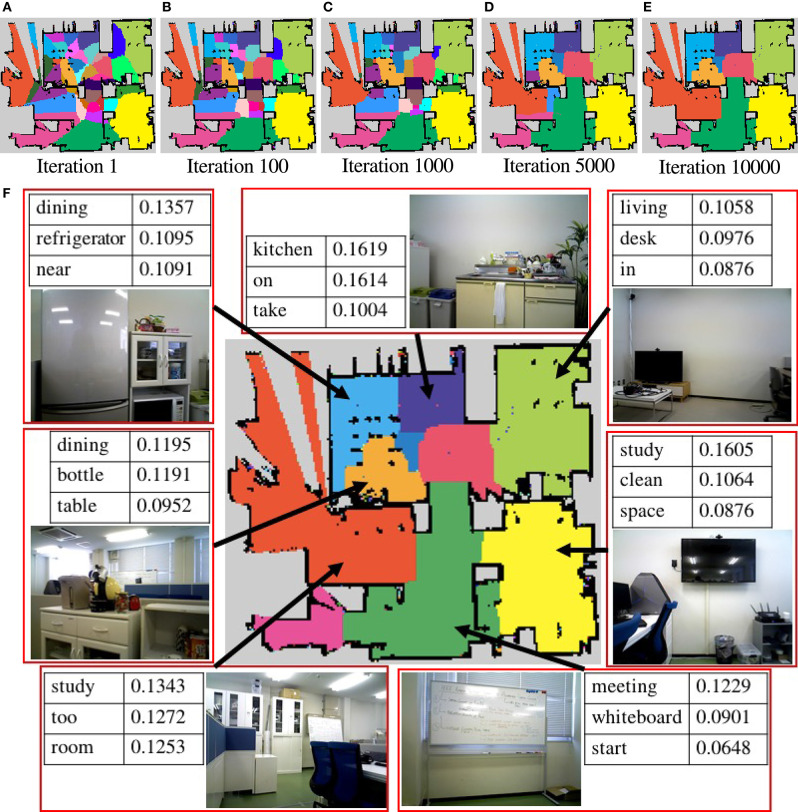
**(A–E)** The semantic maps generated by SpCoMapping in each iteration. Each color represents a different spatial concept. **(F)** The semantic map of the final iteration, with the three best words obtained for the representative spatial concept and some examples of image given at that place. The number in the tables represents the word probability of the spatial concept.

The results of the generated semantic maps of each iteration are shown in Figures 10A–E. The semantic map of the pre-learning by spatial concept formation, shown in Figure 9B, does not deal with environment shape but can categorize a map for some categories. It helps to calculate the parameter of the multinomial distribution π for MRF in an early iteration. There are many categories in the center of the environment in the semantic maps of iterations 1 and 100, and some categories do not deal with the shape of the environment. However, in the semantic map of iteration 10000, some categories are combined, and each region has an arbitrary shape related to the shape of the environment. Therefore, it is shown, qualitatively, that SpCoMapping can gradually estimate semantic maps even in a real-world environment. In addition, since SpCoMapping uses word information as multimodal data, it can obtain words as features of space without the place name being set by the user. The semantic map of the final iteration, with three best words obtained for the representative spatial concept, is shown in Figure 10F. The robot acquired some vocabulary for place names along with the probability of their occurrence. For example, this result shows that if the robot catches the words “meeting” and “start,” it moves to the front of the whiteboard. However, since the robot used weights by mutual information for vocabulary, meaningless words such as “on,” “in,” and “too” have a high probability in the categories of each result. This problem can be resolved by assigning more vocabulary features for the robot to learn.

## 6. Conclusions

This paper proposed a novel semantic mapping method called SpCoMapping extended a spatial concept acquisition method using MRF. Experiments showed that SpCoMapping could deal with the problems faced by existing semantic mapping methods. The semantic maps that SpCoMapping generated in a simulation environment matched those generated by a participant more accurately compared to the existing methods. Furthermore, the semantic maps generated by SpCoMapping are better than those generated by the existing methods from the viewpoint of estimating place from word input, i.e., from the viewpoint of human–robot communication. Finally, an experiment in a real-world daily environment showed that SpCoMapping could generate a semantic map in a real environment as well as a simulated one. SpCoMapping can generate semantic maps dealing with the shape of the environment, and the robot can perform the task including place names. SpCoMapping with Dirichlet prior is more stable than SpCoMapping with DP prior. Therefore, we will use SpCoMapping with Dirichlet prior when we can use the number of semantic categories. However, it is rare when we can use the number of categories in a complex real-world daily environment, so we will use SpCoMapping with DP prior because SpCoMapping with DP prior estimates the number of semantic categories simultaneously.

In future work, we will apply the proposed method to tasks such as those executed by autonomous vacuum cleaner robots, e.g., “please clean my room,” that require communication with humans. Improving the stability of SpCoMapping (DP), as mentioned in the simulation experiment, is also a future challenge. In addition, SpCoMapping employs batch learning; therefore, we will also investigate the development of an online learning algorithm for SpCoMapping and integrating it with SLAM to work in new environments. In this paper, we proposed a 2D semantic mapping method. However, when a robot grasps or detects an object, it will need 3D semantic maps. Therefore, we will also extend this method to 3D. SpCoMapping can deal with the shape of an environment using MRF, however, the environment shape must have features, for example, the corridor is narrow or the entrance connects two spaces. Therefore, we will use a generative adversarial network or a variational autoencoder in order to generate map features.

Although several challenges remain, our proposed method significantly improved the performance of unsupervised learning-based semantic mapping, enabling a robot to make use of users' utterances in a daily environment for semantic mapping. We believe this method will contribute to learning-based human-robot semantic communication in daily environments in the near future.

## Author Contributions

YK designed the study, and wrote the initial draft of the manuscript. All other authors contributed to analysis and interpretation of data, and assisted in the preparation of the manuscript. All authors approved the final version of the manuscript, and agree to be accountable for all aspects of the work in ensuring that questions related to the accuracy or integrity of any part of the work are appropriately investigated and resolved.

### Conflict of Interest Statement

The authors declare that the research was conducted in the absence of any commercial or financial relationships that could be construed as a potential conflict of interest.

## Footnotes

^1^Agent simulator SIGVerse (Version 3) http://www.sigverse.org/wiki/en/.

^2^Code is available at: https://github.com/EmergentSystemLabStudent/spco_mapping.git.

^3^Dataset is available at: https://github.com/EmergentSystemLabStudent/Dataset_of_SpCoMapping.git.
